# TGF-β Isoform Specific Regulation of Airway Inflammation and Remodelling in a Murine Model of Asthma

**DOI:** 10.1371/journal.pone.0009674

**Published:** 2010-03-12

**Authors:** Stephen E. Bottoms, Jane E. Howell, Alistair K. Reinhardt, Iona C. Evans, Robin J. McAnulty

**Affiliations:** Lung Pathobiology Group, Centre for Respiratory Research, University College London, London, United Kingdom; University of Pittsburgh, United States of America

## Abstract

The TGF-β family of mediators are thought to play important roles in the regulation of inflammation and airway remodelling in asthma. All three mammalian isoforms of TGF-β, TGF-β_1–3_, are expressed in the airways and TGF-β_1_ and -β_2_ are increased in asthma. However, there is little information on the specific roles of individual TGF-β isoforms. In this study we assess the roles of TGF-β_1_ and TGF-β_2_ in the regulation of allergen-induced airway inflammation and remodelling associated with asthma, using a validated murine model of ovalbumin sensitization and challenge, and isoform specific TGF-β neutralising antibodies. Antibodies to both isoforms inhibited TGF-β mediated Smad signalling. Anti-TGF-β_1_ and anti-TGF-β_2_ inhibited ovalbumin-induced sub-epithelial collagen deposition but anti-TGF-β_1_ also specifically regulated airway and fibroblast decorin deposition by TGF-β_1_. Neither antibody affected the allergen-induced increase in sub-epithelial fibroblast-like cells. Anti- TGF-β_1_ also specifically inhibited ovalbumin-induced increases in monocyte/macrophage recruitment. Whereas, both TGF-β_1_ and TGF-β_2_ were involved in regulating allergen-induced increases in eosinophil and lymphocyte numbers. These data show that TGF-β_1_ and TGF-β_2_ exhibit a combination of specific and shared roles in the regulation of allergen-induced airway inflammation and remodelling. They also provide evidence in support of the potential for therapeutic regulation of specific subsets of cells and extracellular matrix proteins associated with inflammation and remodelling in airway diseases such as asthma and COPD, as well as other fibroproliferative diseases.

## Introduction

Asthma is characterised by bronchial hyperreactivity, chronic inflammation and airway remodelling [1], with excess subepithelial deposition of extracellular matrix (ECM) molecules including collagens and proteoglycans [2]–[6], that correlates with increased fibroblast/myofibroblast number [4], [7], [8], airway hyperresponsiveness [9], and reduced lung function [10]. The mechanisms responsible for the pathologic features of asthma are incompletely understood. However, they are generally considered to involve complex interactions between resident and infiltrating cells and the mediators they produce [11]. One group of mediators thought to be central, are the transforming growth factor-β (TGF-β) polypeptide family.

There are three mammalian isoforms, TGF-β_1–3_, which play important roles in regulating inflammation, cell growth and differentiation, including of ECM metabolism [1]. In the normal human lung, all three isoforms are expressed by and/or localised to the bronchial epithelium, TGF-β_1_ and TGF-β_3_ are expressed by macrophages, and TGF-β_1_ is also expressed by vascular endothelial, smooth muscle and fibroblast-like cells as well as being bound to the sub-epithelial ECM [12]–[18]. In asthmatic airways, *in situ* hybridization and immunohistochemical studies indicate that TGF-β_1_ is increased and associated predominantly with submucosal and inflammatory cells, including fibroblasts, smooth muscle cells, eosinophils, macrophages and the airway ECM, with variable expression associated with epithelial cells [6], [10], [12], [14]–[16], [19], [20]. Increased TGF-β_1_ expression has been attributed predominantly to increases in eosinophils [10], [20] and macrophages [21]. TGF-β_2_ immunostaining has been reported to be increased in the asthmatic epithelium [18] with increased numbers of TGF-β_2_ positive eosinophils and neutrophils in severe asthmatics and mild asthmatics following allergen challenge [22], [23]. In addition, bronchoalveolar lavage (BAL) levels of TGF-β_1_ are elevated basally in asthmatics and both TGF-β_1_ and TGF-β_2_ are increased following allergen challenge [24], [25]. There is little information on TGF-β_3_ although available evidence suggests no difference between controls and asthmatics [22], [23]. There is also evidence for enhanced signalling for TGF-β family members with increased phosphorylated Smad 2/3 [26] and decreased Smad 7 [27] immunoreactivity.

Similar patterns of TGF-β isoform expression have been observed in the mouse lung [13], [28], [29]. Animal models of asthma have shown increased BAL and tissue levels of TGF-β_1_ [30], [31] but there is little information on TGF-β_2_ and TGF-β_3_. As in asthma, allergen challenge in mice is associated with Smad 2/3 activation [32]. Together these data suggest potentially important roles for TGF-β in airway inflammation and remodelling. Indeed, inhibition of TGF-β_1_ or all TGF-β isoforms modulates responses to allergen sensitisation and challenge [31], [33]–[36] but the conclusions have not been consistent between studies, possibly due to differences in allergen, species or the selectivity of inhibitory approaches.

Data from TGF-β isoform-specific knockout mice demonstrate distinct non-redundant roles for the three TGF-β isoforms in the lung [37]–[39]. However, their relative importance and specific roles in airway inflammation and remodelling are unknown. In this study we utilise isoform specific neutralizing antibodies to assess the roles of TGF-β_1_ and TGF-β_2_ in inflammation and deposition of airway subepithelial ECM molecules using a previously validated mouse model of ovalbumin (OVA) sensitization and challenge [40]. Isoform specific neutralising antibodies reduced TGF-β signalling in the airways and revealed novel isoform-specific and -shared roles in the regulation of airway inflammation and remodelling.

## Methods

### Ethics Statement

Animal studies were approved by the UCL Biosciences Ethical Review Committee and experiments carried out under appropriate UK Home Office approved licence in accordance with the Animals (Scientific Procedures) Act 1986. Animals were maintained in a controlled environment which included filtered air and a 12 hour light/dark cycle. All animals had free access to food and water.

### Animal studies

Ovalbumin sensitisation and challenge was carried out using previously validated adjuvant free methods shown to result in increased OVA specific IgE levels, airway hyperresponsiveness, eosinophilic inflammation, goblet cell hyperplasia and persistent airway remodelling [40], [41]. SV129/C57BL/6 mice were bred at University College London from breeding pairs obtained from the Jackson Laboratory. Briefly, 2–3 month old mice were sensitised by i.p. injection of 10 µg chicken ovalbumin (grade V, Sigma-Aldrich) in 0.1 ml normal saline or saline alone on two occasions 10 d apart. 21 d after the second sensitisation, mice were challenged with 6 daily intratracheal instillations of 400 µg OVA in 50 µl normal saline or saline alone under light halothane anaesthesia. Since previous studies had shown that there were no inflammatory or remodelling effects in saline sensitised/OVA challenged or OVA sensitised/saline challenged animals, these control groups were omitted from this study [40]. Previously characterised neutralising antibodies to TGF-β_1_ (CAT-192) [42], TGF-β_2_ (CAT-152) [43], [44] or irrelevant IgG control antibodies (CAT-001) provided by MedImmune (formerly Cambridge Antibody Technology) and Genzyme were injected i.p. (5 mg/kg in 0.1 ml PBS) 1d prior to the first OVA challenge and on alternate days up to and including 46d. Antibody dosing was based on a previously reported effective strategy [44]. PBS vehicle was injected in controls. Mice were killed 3, 7 or 12d after the final challenge by i.p. injection of 0.1 ml Euthatal (200 mg pentobarbitone sodium/ml). A longitudinal ventral incision was made in the abdomen and the major vessels were sectioned. Lungs were either lavaged *in situ* or removed and wax embedded as described below to assess airway inflammation and remodelling. Each group contained 6–14 animals and is indicated in the figure legends.

### Tissue preparation and staining

The lungs were inflation fixed by tracheal instillation of 4% paraformaldehyde at 20 cm pressure, the trachea was ligated, and the thoracic contents removed and immersed in fixative for 4 h at 4°C prior to transfer into 15% sucrose in PBS at 4°C overnight. The lungs were rinsed in 50% ethanol/H_2_O and stored in 70% ethanol/H_2_O prior to dehydration and wax embedding [40].

### Histochemical staining

Lung sections (3 µm) were stained using modified Martius Scarlet Blue (MSB) trichrome or haematoxylin and eosin for the localisation of collagen and assessment of inflammatory cell infiltration respectively.

### Immunohistochemistry

Immunohistochemistry was performed on paraffin embedded sections (5 µm) of mouse lung using antibodies to TGF-β_1_ (sc-146; Santa Cruz Biotechnology), TGF-β_2_ (sc-90), TGF-β_3_ (sc-82), pSmad 2/3 (cs3101; Cell Signaling Technology), decorin (LF-113; kindly provided by Dr L.W. Fisher, National Institutes of Health) and F4/80 (MCA497G; AbD Serotec). Sections were dewaxed, rehydrated and antigen retrieval achieved by either proteinase K digestion (20 µg/ml) for 10 minutes at room temperature (TGF-β_1_, -β_2_, -β_3_) or by microwaving in 10 mM citrate buffer, pH 6, for 10 minutes (pSmad 2/3 and decorin). Sections were washed in TBS and endogenous peroxidase blocked with 3% hydrogen peroxide (Sigma-Aldrich) for 30 minutes at room temperature. Sections were then washed in TBS and incubated with 4% (v/v) goat serum (DakoCytomation) for TGF-β_1_, -β_2_, -β_3_ and decorin antibodies or rabbit serum (DakoCytomation) for F4/80 antibodies for 20 minutes at room temperature to block non-specific binding sites. Excess serum was removed and the sections incubated overnight at 4°C with primary antibodies at pre-optimised dilutions. The sections were washed and incubated with 0.5% (v/v) goat anti-rabbit or rabbit anti-rat biotin-labelled secondary antibodies (DakoCytomation) as appropriate for 60 minutes at room temperature. Further washes in TBS were performed before the sections were incubated with 0.5% streptavidin (DakoCytomation) for 30 minutes at room temperature. Excess streptavidin was removed by washing in TBS and the sections were treated with DAB (Vector Laboratories) for 10 minutes. They were then washed in distilled water, counterstained in Mayer's haematoxylin, differentiated in acid alcohol, washed in tap water, dehydrated, cleared in xylene and mounted. Negative controls included use of isotype IgG in place of primary antibody or pre-incubation of primary antibody with blocking peptide at 6x the primary antibody concentration.

### Image analysis

The area of airway subepithelial staining for collagen and decorin was estimated using established methods [40]. Briefly, sections were examined by light microscopy using a x10 objective. Airways for analysis were selected using the following predefined criteria. Suitable airways were: complete, of an appropriate size to be contained within a high power field, not attached to other airways and cut in a plane perpendicular to their length (the minimum internal diameter: maximum internal diameter ratio was more than 0.5 in all cases). All suitable airways in each section were analysed. Images were captured with a digital video camera with a resolution of 1392×1040 pixels (QICAM 12-bit color Fast 1394) and QCapture Pro 6.0 software (Media Cybernetics). Pixel size was converted to micrometres and image analysis performed using SimplePCI 6 software (Hamamatsu Corp). The airway lumen perimeter for each suitable airway was measured. Thresholding was carried out using predefined RGB criteria for extracellular matrix components, pixels not adjoining at least 10 others were deleted, providing good definition of the airway matrix. Staining area was calculated for each airway and results expressed as area of sub-epithelial matrix/unit airway perimeter (µm^2^/µm).

To confirm the efficacy of TGF-β neutralising antibodies, numbers of airway epithelial cells with nuclear localised phosphorylated Smad 2/3 per 1000 µm airway lumen perimeter were quantified and expressed as a percentage.

### Bronchoalveolar lavage (BAL)

Following laparotomy and exsanguination, the trachea was cannulated with a 22-gauge venflon (Ohmeda BOC) and the lungs lavaged with 5 ml of PBS in 10×0.5 ml aliquots as described previously [45]. The BAL fluid was kept on ice throughout the procedure and more than 90% of the instilled volume was consistently recovered. BAL samples were centrifuged (300×g) at 4°C for 5 minutes to pellet the cells and the fluid removed. The cell pellet was re-suspended in 500 µl of DMEM containing 10% FBS. Total BAL cell numbers were determined using a haemocytometer. Cytospins of BAL were prepared by centrifuging 0.5−1×10^6^ cells/ml onto poly-L-lysine coated slides (Cytospin 3; Shandon). The slides were air-dried, fixed in methanol and stained using DiffQuik (Dade AG). At least 500 cells per sample were differentiated, using conventional morphological criteria for macrophages/monocytes, lymphocytes, eosinophils and neutrophils and expressed with respect to total cells recovered.

### Assessment of fibroblast decorin production

Primary mouse lung fibroblasts[45] were seeded onto glass coverslips in 24 well plates at a density of 20,000 cells/well in DMEM containing 20% FBS and allowed to adhere. After 24 h the media was replaced with fresh serum free medium with or without TGF-β_1_ or -β_2_ (0.05–1 ng/ml, R&D Systems) for 48 h. The media was then removed, the cell monolayer washed, air-dried and fixed in methanol. The cells were immunostained for decorin as described above.

### Statistical analysis

Data are presented as means ± SEM. Statistical evaluations were performed by ANOVA and Tukey-Kramer post test for multiple comparisons or unpaired t-tests for single comparisons using GraphPad Prism (GraphPad Software). P-values of less than 0.05 were considered significant.

## Results

### TGF-β isoform localisation in normal lung and remodelling mouse airways

The localisation of TGF-β isoforms following OVA sensitisation and challenge, was assessed at 3, 7 and 12 d following final challenge, using pre-validated isoform selective antibodies.

### TGF-β_1_

**Figure 1:**
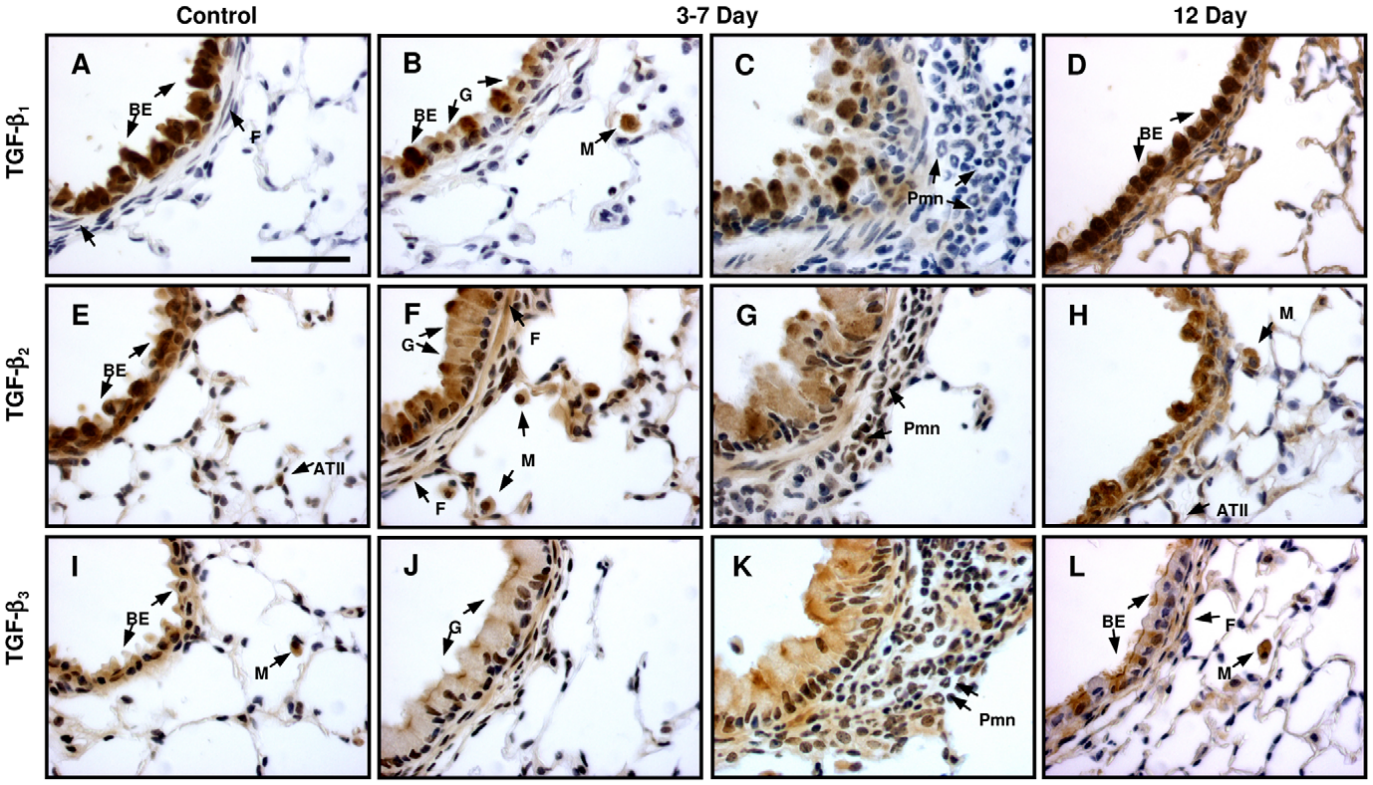
Localisation of TGF-β_1_, -β_2_ and -β_3_ in normal and OVA challenged mouse airways. Representative images from control lungs (A, E and I) and OVA sensitised and challenged mice 3–7 d (B, C, F, G, J and K) and 12 d (D, H and L) after final OVA challenge, immunostained (brown) for TGF-β_1_ (A-D), TGF-β_2_ (E-H) and TGF-β_3_ (I-L). Different cell populations are indicated by arrows; BE - bronchial epithelial cell, F - fibroblast-like cell, G - goblet cell, M - macrophage, Pmn - polymorphonuclear cell, ATII - type II alveolar epithelial cell. Representative images are shown from n = 6–8 animals per experimental group. Scale bar represents 50 µm.

In control lung TGF-β_1_ was associated predominantly with the bronchial epithelium (Fig. 1A) and occasional macrophages. Smooth muscle, endothelial and mesothelial cells, as well as the sub-epithelial ECM were weakly stained. Occasional fibroblast-like cells and type II alveolar epithelial cells (AECs) were also stained. Three and seven days following final ovalbumin challenge goblet cell hyperplasia was observed which stained only weakly for TGF-β_1_ whilst the reduced numbers of bronchial epithelial cells remained strongly stained (Fig. 1B). In addition, there were increased numbers of positively stained macrophages (Fig. 1B). Increased numbers of polymorphonuclear cells (PMNs) were present but were predominantly negative for TGF-β_1_ staining (Fig. 1C). In addition, TGF-β_1_ staining of the sub-epithelial ECM was weak to moderate. By 12d post challenge, goblet cell numbers had declined and bronchial epithelial staining for TGF-β_1_ was strong and similar to controls (Fig. 1A and D). Macrophages remained strongly stained and at this time some PMNs were positive. The subepithelial ECM was also more intensely stained (Fig. 1D).

### TGF-β_2_

Staining for TGF-β_2_ in control airways (Fig. 1E) was similar to that for TGF-β_1_ although smooth muscle cells stained more intensely compared with other cell populations and fibroblast-like cells stained more consistently. In three and seven day post challenge animals, goblet cells showed moderate, uniform staining for TGF-β_2_ whilst bronchial epithelial cells maintained strong staining although less intense and less numerous than in controls (Fig. 1F). In contrast to TGF-β_1_, PMNs showed mixed but predominantly positive staining for TGF-β_2._ Macrophages were positive, sub-epithelial fibroblast-like cells showed mixed moderate to negative staining and the subepithelial ECM demonstrated weak to moderate staining (Fig. 1F). Likewise at 12d, the majority of PMNs and macrophages (Fig. 1H) present were strongly stained. Bronchial epithelial cells were stained for TGF-β_2_ (Fig. 1H) but less intensely than in controls. Fibroblast-like cells again showed mixed positivity and in some areas peribronchial type II AECs were strongly stained (Fig. 1H).

### TGF-β_3_

TGF-β_3_ staining was also predominantly associated with bronchial epithelial cells in control lung although not all cells were stained (Fig. 1I). Macrophages and smooth muscle cells were prominently stained but staining of other cell populations which stained positively for TGF-β_1_ and TGF-β_2_ were only sporadically and weakly stained for TGF-β_3_. Three to seven days after final challenge showed weak, diffuse staining of goblet cells (Fig. 1J) with epithelial staining returning towards control levels by 12d (Fig. 1L). Macrophages were generally stained, PMNs and subepithelial fibroblast-like cells showed mixed but predominantly positive staining (Fig. 1K) at all time-points. In contrast to TGF-β_1_ and TGF-β_2,_ TGF-β_3_ staining of subepithelial ECM was weak at all times.

### Inhibition of TGF-β activity reduces TGF-β signalling via the Smad pathway

**Figure 2:**
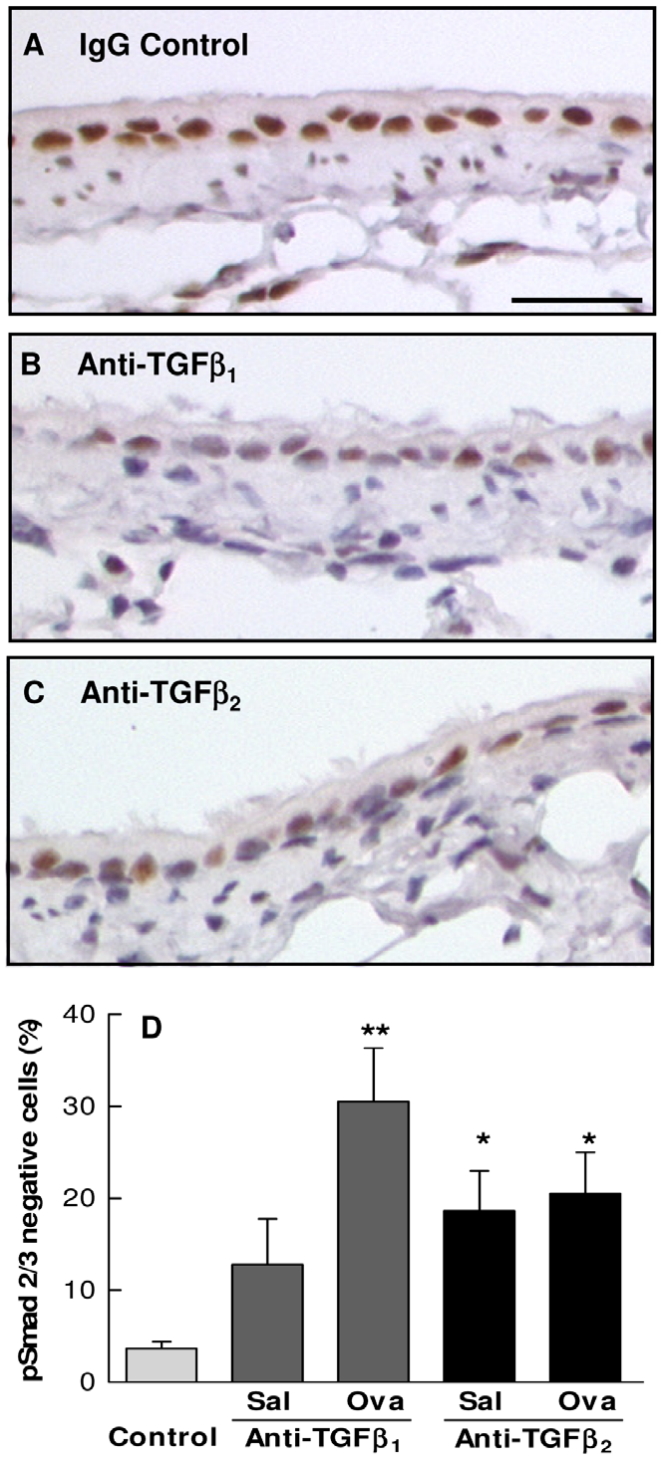
Treatment with isoform specific TGF-β antibodies inhibits signalling via the Smad pathway. (A-C) Representative images of airways stained for phosphorylated Smad 2/3 (brown) from saline sensitised and challenged mice treated with irrelevant IgG (A), anti-TGF-β_1_ (B) or anti-TGF-β_2_ (C) 12 days after final challenge. Phosphorylated Smad 2/3 localises to the nucleus. Note the reduction in intensity of staining and number of positive cells in the airways of animals treated with TGF-β antibodies (B-C) compared with controls (A). Scale bar represents 25 µm. (D) Quantification of the proportion of bronchial epithelial cells negative for phosphorylated Smad 2/3 in IgG controls and saline (Sal) or ovalbumin (Ova) sensitised and challenged animals treated with TGF-β antibodies. Each value represents the mean ± SEM of measurements from 9–10 animals per group. * P<0.05 or ** P<0.001 compared with control.

**Figure 3:**
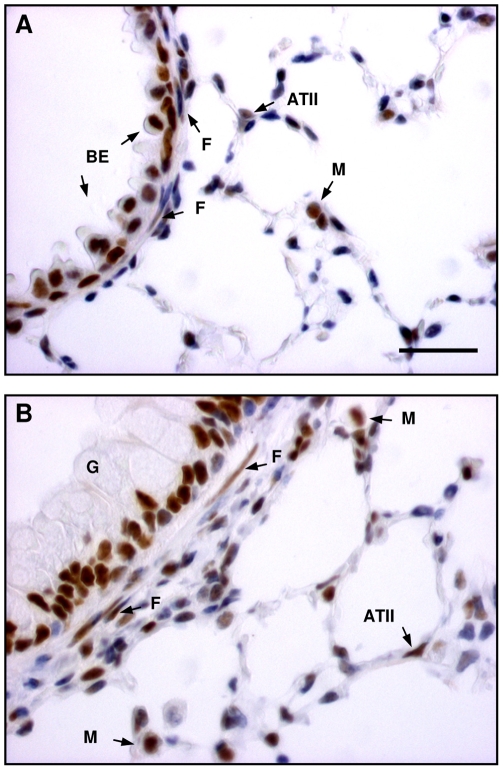
TGF-β signalling in remodelling mouse airways. Phosphorylated Smad 2/3 staining of control (A) and remodelling (B) mouse airway 3 days following final challenge. Cells showing active TGF-β signalling via Smad 2/3 indicated by brown nuclear staining include bronchial epithelial cells (BE), fibroblast-like cells (F), macrophages (M) and type II alveolar epithelial cell (ATII). Scale bar represent 25 µm.

To confirm the activity of isoform specific TGF-β antibodies, lung sections from animals 12d following final challenge were immunostained for phosphorylated Smad 2/3. Control lung sections showed strong nuclear localisation of staining, associated predominantly with bronchial epithelial cells and occasional subepithelial fibroblast-like cells in the airway sub-epithelial layer (Figs. 2A and 3A). Staining was also prominent in type II and some type I AECs, and macrophages (Fig. 3A). In lungs from saline and ovalbumin sensitised and challenged animals treated with neutralising antibodies to TGF-β_1_ or TGF-β_2_ staining intensity was greatly reduced or absent in a greater proportion of cells compared with control lungs (Fig. 2A-D). Together these data suggest that the antibodies to TGF-β_1_ and TGF-β_2_ attained sufficient concentrations in the lung to inhibit TGF-β signalling.

### TGF-β signalling in the remodelling airway

pSmad 2/3 immunostaining was also used to examine changes in TGF-β signalling in allergen challenged airways. Following OVA sensitization and challenge a marked goblet cell hyperplasia was observed at three to seven days and these cells did not stain for pSmad 2/3, however, the basal airway epithelial cells remained strongly positive (Fig. 3B). Peribronchial macrophages were strongly positive and there was an increase in the number of spindle shaped subepithelial fibroblast-like cells which showed mixed staining (Fig. 3B). Infiltrating PMNs were generally negative.

### Effect of TGF-β isoform specific inhibition on sub-epithelial collagen deposition

**Figure 4:**
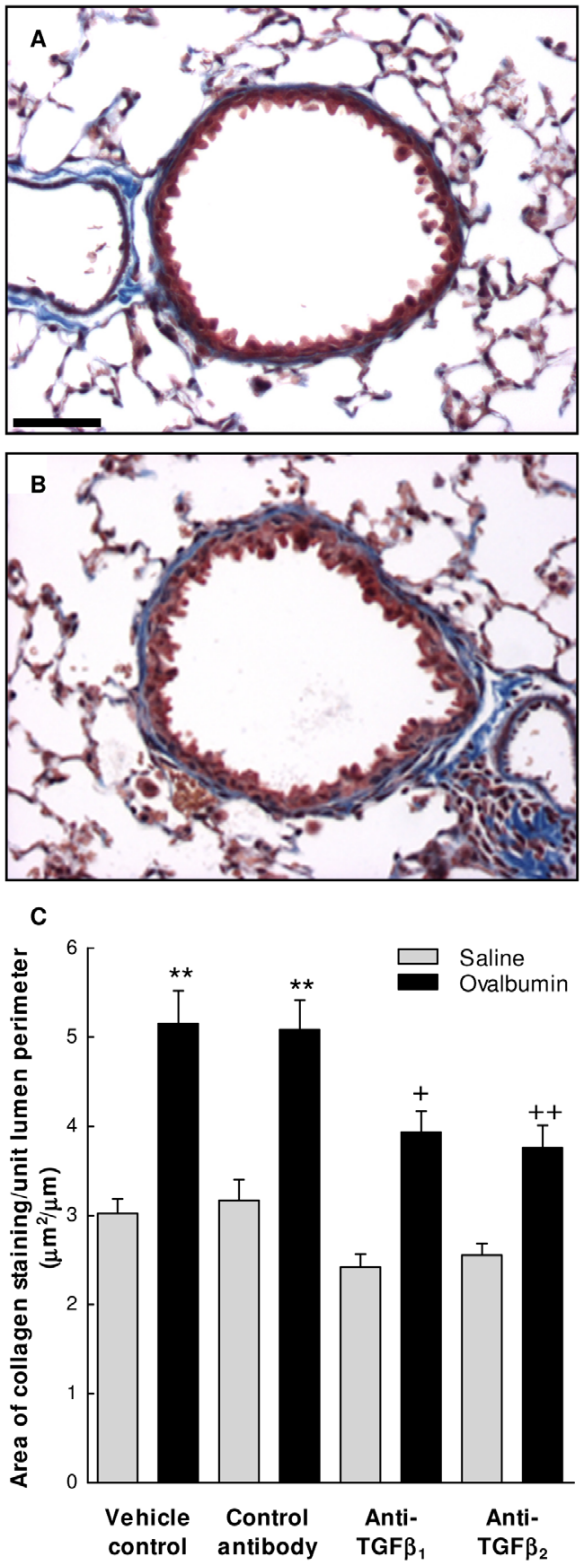
TGF-β isoform shared regulation of allergen-induced sub-epithelial collagen deposition. Control (A) and OVA sensitised and challenged (B) mouse airways 12 days following final challenge demonstrating allergen-induced increase in sub-epithelial collagen deposition depicted by blue staining of the thickened airway wall using a modified Martius Scarlet Blue stain. Scale bar represents 50 µm. (C) Quantification of the area of collagen staining demonstrating OVA-induced increase in sub-epithelial collagen deposition and its inhibition by treatment with antibodies to TGF-β_1_ or -β_2_. Each value represents the mean ± SEM of measurements from 7–10 animals per group. **, P<0.001 compared with relative control. ^+^, P<0.02 and ^++^, P<0.01 compared with OVA control groups.

To assess the effect of TGF-β isoform specific inhibition on OVA-induced subepithelial collagen deposition 12d following final challenge, the area of airway sub-epithelial MSB staining was measured (Fig. 4) using previously validated methods[40]. In vehicle control animals the area of airway sub-epithelial collagen was 3.02±0.17 µm^2^/µm lumen perimeter and this increased by approximately 70% (p<0.001) in OVA exposed mice (Fig. 4C). Treatment of animals with an IgG control antibody did not affect basal (saline sensitised and challenged animals) or OVA-induced collagen deposition. Similarly treatment with specific antibodies to TGF-β_1_ or TGF-β_2_ did not significantly affect basal levels of collagen. Antibodies to TGF-β_1_ or TGF-β_2_ inhibited the OVA-induced increase in sub-epithelial collagen deposition by approximately 60% and 70% compared with the control group (p<0.02 and p<0.01 respectively). Furthermore, the area of sub-epithelial collagen in the airways of OVA exposed, anti-TGF-β_1_ or anti-TGF-β_2_ treated animals was not significantly increased above that observed in saline sensitised and challenged control animals.

### Effect of TGF-β isoform specific inhibition on sub-epithelial decorin deposition

**Figure 5:**
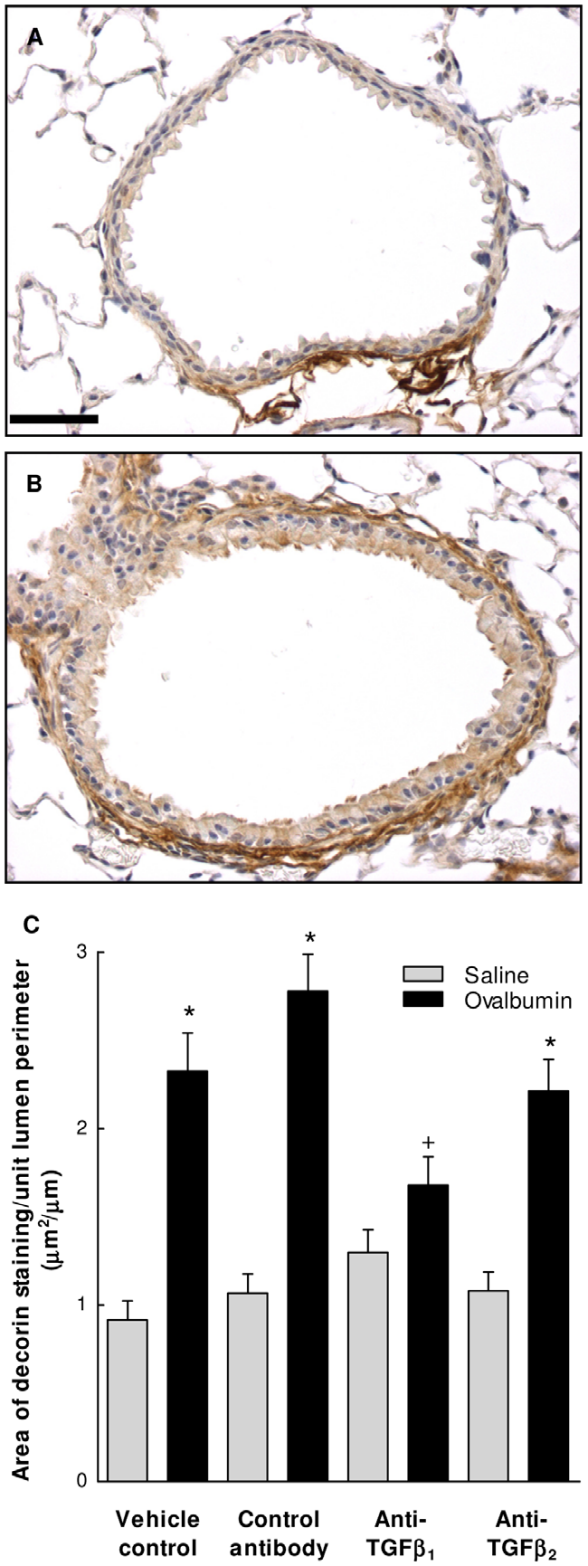
TGF-β isoform specific regulation of allergen-induced sub-epithelial decorin deposition. Control (A) and OVA sensitised and challenged (B) mouse airways 12 days following final challenge demonstrating allergen-induced increase in sub-epithelial decorin deposition depicted by brown staining of the thickened airway wall by immunohistochemistry. Scale bar represents 50 µm. (C) Quantification of the area of decorin staining demonstrating OVA-induced increase in sub-epithelial decorin deposition and its inhibition by treatment with antibodies to TGF-β_1_ but not TGF-β_2_. Each value represents the mean ± SEM of measurements from 9–10 animals per group. *, P<0.001 compared with relative control. ^+^, P<0.01 compared with OVA control and not significantly different to the saline anti-TGF-β_1_ group.

To investigate whether ECM protein deposition is co-ordinately regulated by TGF-β_1_ and TGF-β_2_ in airway remodelling, changes in sub-epithelial decorin deposition were also examined (Fig. 5). In controls, decorin deposition around the airways was less prominent with areas of staining approximately one-third that of collagen (0.92±0.11 µm^2^/µm lumen perimeter). Following OVA sensitization and challenge decorin deposition increased by approximately 2.5 fold (p<0.001) 12 days following final challenge. Treatment of animals with an IgG control antibody did not affect basal or OVA-induced decorin deposition and treatment with specific antibodies to TGF-β_1_ or TGF-β_2_ also had no effect on basal levels of sub-epithelial decorin. Treatment of animals with anti-TGF-β_1_ inhibited the OVA-induced increase in decorin deposition by approximately 50% (p<0.01) with values not significantly raised above those observed in saline controls. In contrast to the effects on collagen, antibodies to TGF-β_2_ had no effect on OVA-induced sub-epithelial decorin deposition.

### Effect of TGF-β_1_ and TGF-β_2_ on production of decorin by murine lung fibroblasts

**Figure 6:**
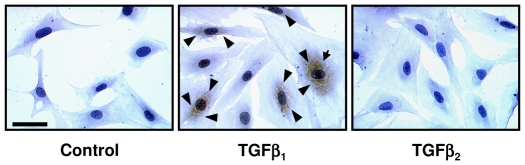
Effect of TGF-β isoforms on murine lung fibroblast decorin production. Mouse lung fibroblasts were incubated with or without TGF-β isoforms for 48 h and decorin production assessed by immunocytochemistry. Control cells and cells incubated with TGF-β_2_ showed little or no staining for decorin. In contrast cells incubated with TGF-β_1_ showed increased decorin staining (brown) localised to the peri-nuclear cytoplasm (arrows). Scale bar represents 50 µm.

To further verify the selective effect of TGF-β_1_ in the regulation of decorin production, primary mouse lung fibroblasts were cultured with TGF-β_1_ or TGF-β_2_ and their effect on decorin production was assessed by immunocytochemistry. In control untreated fibroblasts little or no decorin staining was detected. However, consistent with the *in vivo* data, fibroblasts incubated in the presence of TGF-β_1_ showed an increase in cell associated decorin with maximal effects at 0.25–0.5 ng/ml (Fig. 6). Whereas incubation with TGF-β_2_, up to 1 ng/ml, showed no apparent increase in decorin staining, providing further evidence that decorin synthesis is selectively regulated by the TGF-β_1_ isoform.

### Effect of TGF-β isoform specific inhibition on ovalbumin-induced increase in sub-epithelial fibroblast-like cells

Staining of lung sections for pSmad 2/3 highlighted the presence of increased numbers of sub-epithelial spindle-shaped fibroblast-like cells (Fig. 3). Quantification showed that the numbers of these cells almost doubled in OVA sensitised and challenged animals compared with controls (10.79±0.36 vs. 19.72±0.55 cells/mm lumen perimeter; p<0.001). Inhibition of TGF-β activity with specific antibodies to TGF-β_1_ or TGF-β_2_ did not alter the basal or OVA-induced increase in fibroblast-like cell number.

### Effect of TGF-β isoform specific inhibition on ovalbumin-induced inflammatory cell profiles

**Figure 7:**
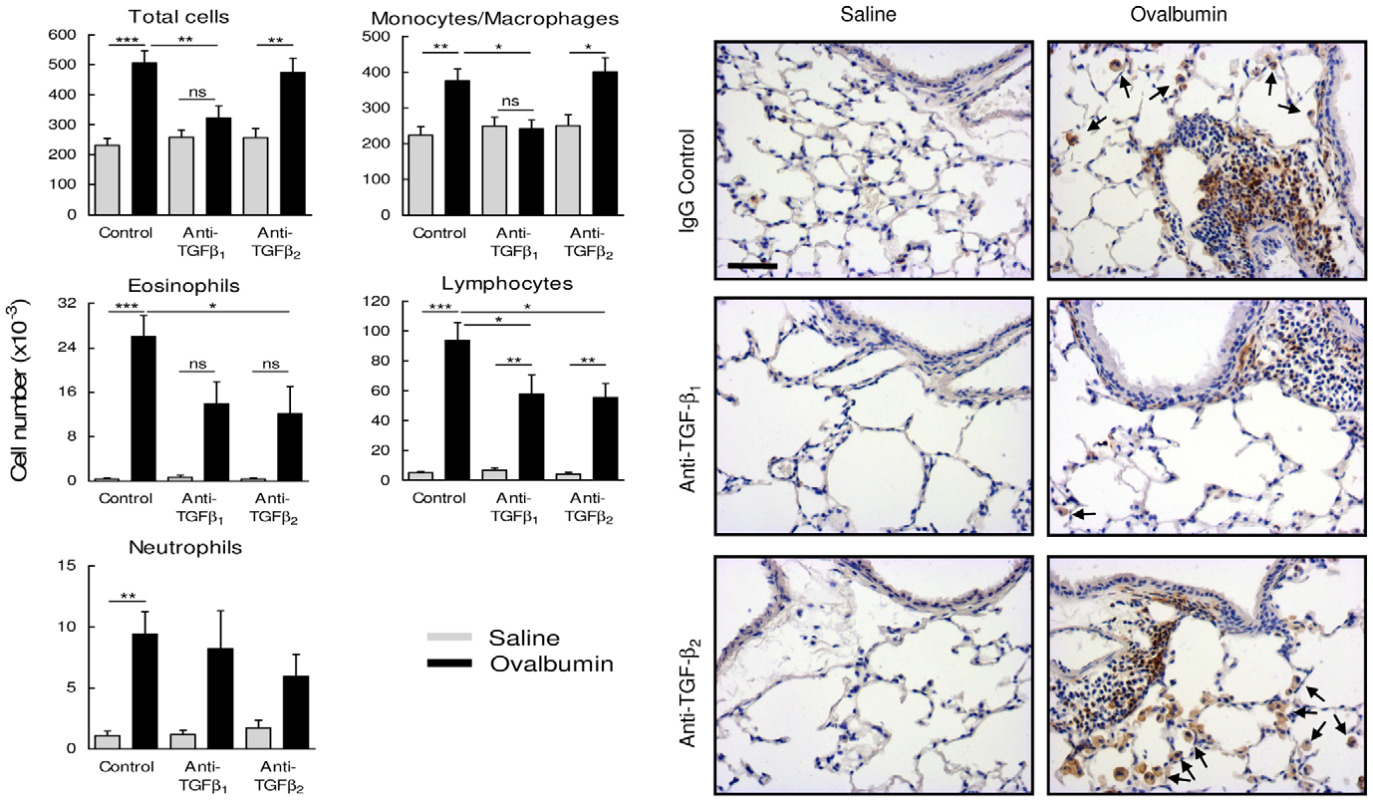
TGF-β isoform shared and selective effects on allergen-induced inflammation. Graphs show total BAL cell numbers and the relative contributions of monocytes/macrophages, eosinophils, lymphocytes and neutrophils isolated from animals 12 days following final challenge. Each bar represents the mean ± SEM of values from 9–14 animals per group. *, P<0.05. **, P<0.01. ***, P<0.001. Photomicrographs show staining of lung sections for the macrophage and macrophage progenitor surface antigen, F4/80, demonstrating inhibition of macrophage accumulation (arrows) around the airways of animals treated with antibodies to TGF-β_1_ compared with sections from animals treated with control IgG or anti-TGF-β_2_. Scale bar represents 50 µm.

To examine changes in airway inflammation we sampled the airway cell population by BAL 12d following final challenge. Total cells recovered from OVA sensitised and challenged animals increased approximately 2.5-fold (p<0.001) compared with controls (Fig. 7). This increase included an approximate 2-fold increase in the number of monocytes/macrophages and much larger fold-increases in the numbers of eosinophils, lymphocytes and neutrophils (Fig. 7). Inhibition of TGF-β_1_ or TGF-β_2_ activity did not affect basal lavage cell profiles. Inhibition of TGF-β_1_ but not TGF-β_2_ activity inhibited the OVA-induced increase in total lavage cells by approximately 70% (p<0.01). This was primarily due to a lack of monocyte/macrophage influx in OVA challenged animals treated with anti-TGF-β_1_. This finding was corroborated by immunohistochemical staining of tissue sections for the macrophage selective cell surface marker, F4/80, which also showed a dramatic reduction in macrophages in peribronchial regions of OVA challenged animals treated with anti-TGF-β_1_ (Fig. 7). In addition, inhibition of TGF-β_1_ or TGF-β_2_ reduced the OVA-induced increase in BAL eosinophils and lymphocytes by approximately 50% (Fig. 7). There was no significant effect of inhibiting TGF-β_1_ or TGF-β_2_ on OVA-induced neutrophil numbers.

## Discussion

**Figure 8:**
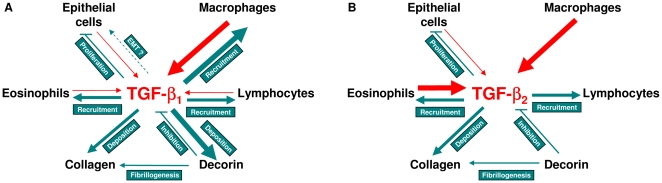
Schematic representation of the source and roles of TGF-β_1_ and TGF-β_2_ in OVA-induced airway inflammation and remodelling. Following OVA sensitisation and challenge there appears to be a shift in the localisation/production of TGF-β_1_ (A) and TGF-β_2_ (B) away from bronchial epithelial cells with increased localisation/production of TGF-β_1_ to macrophages and TGF-β_2_ to macrophages and eosinophils. TGF-β_1_ and TGF-β_2_ are potent inhibitors of bronchial epithelial proliferation, the reduced localisation of TGF-βs to these cells may be directed towards epithelial proliferation and repair. However, greater levels of inflammatory cell derived TGF-β may counteract this. TGF-β_1_ is capable of inducing epithelial-mesenchymal transition of bronchial epithelial cells *in vitro* but whether this occurs in this model or asthmatic airways is uncertain. TGF-β_1_ plays a critical role in the recruitment/maintenance of macrophages and the synthesis/deposition of decorin as well as contributing to the recruitment/maintenance of eosinophils, lymphocytes and synthesis/deposition of collagen. TGF-β_2_ appears to have a more restricted role, contributing to the recruitment/maintenance of eosinophils, lymphocytes and the synthesis/deposition of collagen. Red arrows denote synthesis/localisation of TGF-β isoforms, blue illustrates functions of the isoforms based on this study and the literature. The width of the arrows symbolise the apparent relative importance of the source of that isoform or its function.

The TGF-β family of mediators is thought to play important roles in the pathogenesis of asthma associated with the regulation of inflammation and airway remodelling. Although there is evidence that expression of the different TGF-β isoforms changes in asthma there is a lack of information on the specific roles of the individual isoforms. Here, using an animal model and TGF-β isoform specific neutralising antibodies, we show that TGF-β_1_ specifically regulates OVA-induced increases in macrophages and also sub-epithelial deposition of decorin. In contrast, both TGF-β_1_ and TGF-β_2_ were found independently to contribute to OVA-induced increases in eosinophils, lymphocytes and sub-epithelial deposition of collagen (Fig. 8). We further demonstrate that allergen-induced increases in peribronchial fibroblasts, a feature that has previously been correlated with increased levels of TGF-β in asthmatic airways, were not affected by selective inhibition of TGF-β_1_ or TGF-β_2_.

In control lungs, localisation of the three TGF-β isoforms showed broadly similar patterns to those previously described for mouse, rat and human [12]–[14], [16], [17], [28], [29]. Localisation of TGF-β_1_ in OVA challenged animals was similar to that previously reported in asthmatic airways and animal models [4], [10], [16], [18], [18], [22], [30,31] although at early times we found PMNs were mostly negative for TGF-β_1_ and only showed positive staining at 12d. In addition, we present novel data on TGF-β_2_ and TGF-β_3_ localisation in OVA challenged mouse lung. The major differences in localization compared with TGF-β_1_ included a reduction in TGF-β_3_ staining of epithelial cells following allergen challenge, uniform moderate staining of goblet cells for TGF-β_2_ compared with very weak staining for TGF-β_1_ and -β_3_, more consistent staining of PMNs for TGF-β_2_ and -β_3_ as well as more consistent staining of fibroblast-like cells for TGF-β_3_.

These studies highlight differences in expression of the TGF-β isoforms and also a shift in the cellular profile of TGF-β localisation. In the normal airway, all three isoforms are predominantly localised to the bronchial epithelium. Whereas, following OVA sensitisation and challenge there appears to be a shift towards decreased bronchial epithelial staining, with increasing numbers of goblet cells staining primarily for TGF-β_2_, macrophages staining for all three TGF-β isoforms, and PMNs staining primarily for TGF-β_2_ and TGF-β_3._ These changes, although complex, likely impact on the response to OVA sensitisation and challenge. For example, the epithelium is both a major source and target for TGF-β in the normal airway. Reduced levels of epithelial TGF-β staining following OVA may facilitate proliferation and repair, whilst increased TGF-β production by inflammatory cells may counter this, potentially limiting epithelial repair, facilitating epithelial-mesenchymal transition and increasing subepithelial extracellular matrix protein production (Fig. 8). In addition, the observed isoform selective changes in cellular expression also suggested the potential for differential roles for the three isoforms in the regulation of inflammation and tissue remodelling.

Previous studies in which all TGF-β isoforms were inhibited with pan-specific antibodies or Smad 3-deficient mice, have shown inhibition of OVA-induced sub-epithelial collagen deposition [33], [35]. Similar results have been obtained using TGF-β_1_ specific antibodies [31]. Our data, using TGF-β_1_ specific antibodies are consistent with these studies and in addition, show for the first time that antibodies specific for TGF-β_2_ have similar inhibitory effects on OVA-induced sub-epithelial collagen deposition. These data suggest that TGF-β_1_ and -β_2_ are equally important in regulating airway collagen deposition.

There is increasing evidence for decorin playing a role in airway remodelling in asthmatics [3], [5], [16] and animal models [40], [46]. In contrast to the coordinate regulation of collagen deposition by TGF-β_1_ and TGF-β_2_ we show that allergen-induced decorin deposition is selectively regulated by TGF-β_1_ with no apparent role for TGF-β_2_ (Fig. 8). This novel finding was confirmed by *in vitro* fibroblast studies. Decorin has important homeostatic roles, being an important regulator of collagen fibrillogenesis [47], and a natural inhibitor of TGF-β activity [48], [49]. Decorin deficiency has been shown to decrease airway resistance and increase lung compliance [50] but decorin over-expression inhibits the development of lung fibrosis [51], presumably at least partly through its inhibition of TGF-β activity. Thus inhibition of decorin could have potentially beneficial and detrimental effects in asthma depending on the dominant mechanisms of action. Further studies are therefore necessary to determine whether inhibition of decorin would be beneficial in asthma.

TGF-β_1_ expression has previously been correlated with increased numbers of fibroblasts/myofibroblasts in the asthmatic airway wall [19]. In addition, studies with Smad 3 deficient mice showed inhibition of OVA-induced peribronchial fibroblast numbers [35]. However, we found no effect of inhibiting TGF-β_1_ or -β_2_ activity on allergen-induced fibroblast/myofibroblast-like cell number. This suggests that mediators other than TGF-β_1_ and -β_2,_ perhaps including TGF-β_3_ or activin A, which also act via Smad 3, are responsible for recruitment and proliferation of these cells.

The effects of TGF-β on inflammation are complex, having both pro- and anti-inflammatory properties [52]. TGF-β is thought to be involved in the recruitment and activation of inflammatory cells in asthmatics, with further production of TGF-β by these cells contributing to persistant airway inflammation and remodelling [53]. Previous studies blocking TGF-β_1_ or TGF-β non-specifically in OVA models have shown either reduced inflammation [34], no effect on inflammation [31], [33], [35] or increased inflammation [27], [54]. The reasons for the lack of consistency between these studies are unclear but most likely relate to differences in the protocols and approaches to inhibit TGF-β activity. However, in all of these studies inflammation was assessed acutely, 24–72 hours after final challenge. In the present study inflammation was assessed 12 days after the final OVA challenge and as with other studies where inflammation has been assessed at extended periods after final challenge [30] the inflammatory profile was more mononuclear in nature (figure 7) than in acute studies. Inhibition of TGF-β_1_ but not TGF-β_2_ completely blocked the allergen-induced increase in monocytes/macrophages and inhibitors of TGF-β_1_ or -β_2_ reduced allergen-induced increases in eosinophils and lymphocytes at this time. TGF-β_1_ is a chemoattractant for monocytes/macrophages, eosinophils and lymphocytes [55]–[57] suggesting that inhibition may reduce inflammatory cell recruitment. Macrophages, eosinophils and lymphocyte sub-populations, such as Th2 cells and T regulatory cells, produce TGF-β thus inhibition of their recruitment could also limit the allergen-induced increase in TGF-β. Consistent with these data, inhibition of TGF-β_1_ or TGF-β_1_ and TGF-β_2_ have previously been shown to reduce monocyte/macrophage numbers in cutaneous wound healing [58]. In addition, the reduction in the OVA-induced monocyte/macrophage numbers associated with the inhibition of TGF-β_1_ may contribute to the reduced subepithelial deposition of extracellular matrix proteins since previous studies have shown that macrophage depletion inhibits the development of fibrosis in other tissues including liver and lung parenchyma [59], [60].

These studies were carried out with the widely used OVA sensitisation and challenge model. Whilst it is recognised that this does not mimic asthma, it does replicate many of the characteristic features of the disease [61]. In the current study we demonstrate that localisation of TGF-β isoforms in control and OVA exposed airways are generally consistent with currently available data in normal and asthmatic human airways. In addition, we have previously shown airway remodelling observed at 12d in this model persists until at least 28d [40]. Together, these data suggest that the model was appropriate for the current studies.

Although, as far as we are aware, this is the first study to identify TGF-β isoform specific effects in the lung there are earlier precedents both in vitro and in vivo. TGF-β_1_ is an isoform specific regulator of osteodifferentiation [62]. TGF-β_2_ is a specific inducer of hair follicle morphogenesis [63] and of differentiation of cardiac myocytes from embryonic stem cells [64]. TGF-β_3_ specifically regulates palate fusion [65].

The mechanisms responsible for TGF-β isoform specific effects are currently unknown. There are multiple points in the pathway from synthesis, through activation, accessory molecule and receptor binding, to signalling which may lead to isoform specific effects [1]. However, neutralising antibodies act on the active peptide hence the isoform specific effects observed in this study most likely relate to altered dynamics between accessory molecules, such as betaglycan and endoglin, and differences in receptor affinities for the uninhibited isoforms. In addition, TGF-β isoforms are capable of inducing expression of the other isoforms thus inhibition may further alter relative isoform expression. Further studies are required to elucidate these potentially complex mechanisms.

In summary, we have demonstrated for the first time that TGF-β isoforms have a combination of specific and shared roles in the regulation of airway inflammation and remodelling (Fig. 8). This provides evidence in support of the potential for therapeutic regulation of specific subsets of cells and extracellular matrix components in inflammation and remodelling associated with airway disease such as asthma and possibly other inflammatory and fibroproliferative diseases.
